# Clinical Learning Environment at Shiraz Medical School in the Educators’ and Residents’ Viewpoints

**DOI:** 10.5812/ircmj.3621

**Published:** 2013-06-05

**Authors:** Rita Rezaee, Sedigheh Ebrahimi

**Affiliations:** 1 Department of Medical Education, Shiraz University of Medical Sciences, Shiraz, IR Iran; 2 Department of Pediatrics, Shiraz University of Medical Sciences, Shiraz, IR Iran

**Keywords:** Learning, Hospitals, Clinical Learning

## Dear Editor,

Learning environment has been found to be one of the most important factors in determining the success of an effective teaching program (1). The quality of educational atmosphere has been identified to be crucial for effective learning (2). The aim of current study were to identify the important determinants operating in clinical learning environment at Shiraz Medical School and the views of resident about strengths and weaknesses of work environment. The study was conducted in three phases. At first the published medical literature about the attributes of learning environment was reviewed. The perception and experiences of medical specialists about such attributes were explored by Delphi’s technique.

In the second phase, the importance of the each attribute that derived from the initial phase was rated by medical residents on a 5-point Likert-type scale as the important key domain that were found to be effective in clinical learning and what they presumed to be considered in clinical setting. In the third phase the quality of clinical learning environment was evaluated by residents through a self-administered questionnaire including 45 items developed based on the items of clinical teaching characteristics derived from the first phase. The content validity of the questionnaire was established. Cronbach’s alpha used to estimate the reliability was 0.76.

Ten domains were found to be important in effective clinical learning environment through reviewing medical literature. The medical faculties (n = 52) and residents’ (n = 132) views about the importance of such domains were asked through a questionnaire survey. Generally, eight broad attributes were indicated by medical faculties as the key determinants that contribute to an effective clinical teaching including; supervision, role clarity, learning opportunity, work load, social support, physical facilities, work diversity, autonomy. Out of one hundred thirty two residents, 66 completed the questionnaire and the data were available for analysis with return rate of 50%.

Comparison of the mean score of the domains indicated a significant difference between perspectives of the faculties and residents except for supervision (P = 0.088 for supervision, P = 0.006 for social support and P = 0.000 in other domains). Various strengths of correlation between the aspects of conceptual domains were identified. A highest correlation was found between social support and learning opportunities (0.54). The opportunity to apply knowledge by the residents can increase autonomy (correlation coefficient = 0.42).

The residents’ perceptions concerning the quality of the aspects of the learning environment in recent clinical rotations were studied. The mean score obtained for all domains was lower than desirable condition (P = 0.000 in all). This difference was especially significant in the case of workload (P = 0.000) which rated as the least score level (1.73 + 1.11). 81.9% of the residents believed that they have a duty to do along with a lot of clerical and other nonmedical tasks and 57% of them stated the inadequate definition of their duties and ward expectations.53.1% of the residents said they had no sufficient teaching rounds. 68.8% of the respondents reported that they had not been given sufficient supervision and feedback by staff faculties and have been poorly supervised.

More than two thirds of the participants believed in insufficient use of assessment methods. In general, the residents underscored the quality of the present clinical climate compared to desirable condition suggests a need for improvement. The highest correlation was found between social support and learning opportunities (0.54). This may reflect that an efficient social support enables the residents to apply knowledge and skills. The greatest difference between the mean of the groups (1.8) was found for supervision.

In the present study satisfaction with the domain of supervision and social support was low and needs enhancement. This finding was in line with result of a previous study conducted by Dolmans et al. in which students noted that clerkship-sites needed sufficient supervision and feedback (3). Helpful supervisory behaviors include direct guidance on clinical work, linking theory and practice, and offering feedback and role modeling (4, 5). In residents’ attitudes, effective supervision in practice settings and giving frequent and immediate feedback to residents on the quality of their learning appears to be the key to the success of clinical learning. In conclusion, although some aspects of clinical learning environment were indicated well, strengthening the positive aspects and correction of weaknesses represents an effective step towards achieving the high levels of quality and meeting the residents’ expectations.
